# Functional restoration of CD56^bright^ NK cells facilitates immune control via IL-15 and NKG2D in patients under antiviral treatment for chronic hepatitis B

**DOI:** 10.1007/s12072-017-9803-4

**Published:** 2017-06-20

**Authors:** Tao Chen, Lin Zhu, Aichao Shi, Lin Ding, Xiaoping Zhang, Zhenmin Tan, Wei Guo, Weiming Yan, Meifang Han, Jidong Jia, Xiaoping Luo, Detlef Schuppan, Qin Ning

**Affiliations:** 1 Department and Institute of Infectious Disease, Tongji Hospital, Tongji Medical College, Huazhong University of Science and Technology, No 1095, Jiefang Avenue, Wuhan, 430030 China; 2 Liver Research Center, Beijing Friendship Hospital, Capital Medical University, Beijing, 10050 China; 3 Department of Pediatrics, Tongji Hospital, Tongji Medical College, Huazhong University of Science and Technology, Wuhan, 430030 China; 4 Institute of Translational Immunology, University Medical Center and Research Center for Immune Therapy, Johannes-Gutenberg-University, Mainz, Germany; 5 Division of Gastroenterology, Beth Israel Deaconess Medical Center, Harvard Medical School, Boston, MA USA

**Keywords:** Natural killer cells, Chronic hepatitis B, Telbivudine, NKG2D, IL-15

## Abstract

**Background and aims:**

Hepatitis B virus (HBV) is intrinsically immunogenic, with long-lasting immune control in many patients. However, the mechanisms and key cell types underlying effective immune control are incompletely understood.

**Methods:**

We studied the restoration of natural killer (NK) cell numbers and function post antiviral treatment in 52 hepatitis B e antigen (HBeAg)-positive chronic hepatitis B (CHB) patients who received telbivudine (LdT) for 48 weeks. Blood samples were collected at week 0, 12, 24, 36, and 48 and tested for HBV DNA, hepatitis B surface antigen (HBsAg), HBeAg, liver enzymes, and NK cell parameters.

**Results:**

Compared with baseline, the number of peripheral CD3^−^CD56^bright^ NK cells increased significantly from week 24 to 48, especially in patients with baseline alanine transaminase (ALT) two- to fivefold the upper line of normal (ULN) or HBV DNA <9 log_10_ copies/ml. Expression (number and density) of activating receptors NKG2D and NKp46 on CD3^−^CD56^bright^ NK cells was enhanced, while inhibitory receptor NKG2A decreased. Notably, numbers of CD3^−^CD56^bright^ or NKG2D^+^CD3^−^CD56^bright^ NK cells were significantly better restored in patients with HBeAg seroconversion. NK cell activating serum interleukin 15 (IL-15) was significantly increased during LdT treatment, especially in HBeAg seroconverters. LdT significantly enhanced expression of NKG2D and IL-15 in cultures of purified peripheral NK cells from treatment-naïve HBeAg-positive CHB patients.

**Conclusions:**

Functional restoration of CD56^bright^ NK cells via upregulation of IL-15 and NKG2D is a novel activity of LdT and likely other antivirals, independent of its effect on HBV replication. This also demonstrates the importance of host immune restoration in controlling chronic HBV infection.

**Electronic supplementary material:**

The online version of this article (doi:10.1007/s12072-017-9803-4) contains supplementary material, which is available to authorized users.

## Introduction

Hepatitis B virus (HBV) infection is a worldwide health problem. Chronic HBV infection (CHB) is an important risk factor for development of liver cirrhosis and hepatocellular carcinoma [1]. Persistent infection by HBV results from inefficient innate and adaptive immune responses [2]. While remarkable progress has been made in achieving durable viral suppression, including a reduction of hard endpoints such as development of cirrhosis or hepatocellular carcinoma (HCC), these therapies usually fail to achieve sustained immune control and viral elimination [3]. Notably, apart from studies on mainly CD8^+^ T cell responses, the immune mechanisms underlying durable HBV suppression and finally elimination in patients with CHB remain ill defined [4].

Recent studies have shown the importance of innate immunity in the pathogenesis of HBV infection, especially natural killer (NK) cells [5–7]. NK cells control pathogen invasion and replication during early phases of HBV infection, while priming and regulating adaptive immune responses later in the course of acute infection and in chronically infected hosts. NK effector function is controlled by a complex network of signals, prominently via activating or inhibitory receptors.

Nucleotide or nucleoside (NUC)-based small molecules are widely used therapeutics for CHB, causing profound viral suppression. In parallel, the NUCs tenofovir (TDF) and adefovir (ADF) were shown to increase production of tumor necrosis factor alpha (TNF-α), IL-6, IL-10, CCL5 (Rantes), and CCR5 (MIP-1α) in human monocytes by an as yet undefined mechanism [8]. A single study reported enhancement of NK cell interferon (IFN)-γ in patients treated with entecavir (ETV) [9], but another study in patients treated with ADF and lamivudine (LAM) did not confirm these results and instead showed decreased expression of the NK cell effector molecule TRAIL [10]. These data suggest that each NUC may display a unique innate immune modulatory activity, and that observed effects may also be dependent on the individual experimental setup.

Most previous studies were limited to cross-sectional observation during antiviral treatment that ignored the dynamic change of host immunity. We present herein a longitudinal study to show restoration of NK cell numbers and functional activity in patients with CHB on treatment with LdT for 48 weeks. Notably, patients with seroconversion developed increased serum IL-15, which proved central to the activation of NK cells exposed to LdT in vitro. Our results may help improve combination treatment protocols aimed at long-lasting viral suppression and elimination.

## Patients and methods

### Study design and participants

The study enrolled 52 HBeAg-positive CHB patients who had been diagnosed according to the “Chronic Hepatitis B: Update 2007” standards. Baseline patient characteristics are presented in Table 1. All patients were treated with LdT for 48 weeks. Blood samples were collected at baseline at week 12, 24, 36, and 48 of treatment and tested for HBV DNA, HBsAg, HBeAg, ALT, aspartate transaminase (AST), and other routine parameters. The efficacy of LdT treatment during 48 weeks is shown in Supplementary Fig. 1.

**Table 1:** Patient characteristics

Characteristics	Value
Number of patients	52
Age (years)^a^	27.0 (17.0–50.0)^a^
Male gender (*n*, %)	31.0 (57.41)^b^
HBV DNA log_10_ (copies/ml)^a^	8.24 (1.03)^b^ (4.57–9.69)^a^
HBsAg^+^ (%)	100.0
HBsAg log_10_ (IU/ml)^b^	4.11 (0.66)^b^ (1.57–5.08)^a^
HBeAg^+^ (%)	100.0
Serum ALT (IU/l)^b^	162.52 (120.15)^b^

^a^Median (interquartile range)

^b^Mean (standard deviation, SD)

### Fluorometric analysis

Peripheral blood mononuclear cells (PBMC) were prepared from patient blood samples, stained for cell surface and intracellular antigens, and analyzed by flow cytometry, according to standard procedures (BD Pharmingen protocol). Antibodies included PECY5-conjugated anti-CD3 mAb (clone HIT3a), APC-conjugated anti-CD56 mAB (clone B159), and PE-conjugated mAbs directed against the NK receptors NKG2A (clone 131411), NKG2C (clone 134591), NKG2D (clone 149810), NKp30 (clone p30-15), and NKp46 (clone 9E2). CD3^−^CD56^+^ NK cells were gated by flow cytometry (Canto II, BD Pharmingen, Franklin Lakes, NJ, USA) and assayed for the above NK cell receptors.

### Cytokine assays

The Cytometric Bead Array technique was used to detect the level of serum interleukin 15 (IL-15), IL-2, IL-4, IL-6, IL-10, tumor necrosis factor alpha (TNF-α), and interferon gamma (IFN-γ) (Human IL15/Th1/Th2 CBA kit, BD Bioscience, Franklin Lakes, NJ, USA). Serum IL-15 was confirmed by a separate enzyme-linked immunosorbent assay (ELISA) (Human IL-15 Quantikine ELISA Kit, R&D system, Minneapolis, MN, USA).

### Culture of peripheral NK cells with telbivudine/lamivudine in vitro

Peripheral NK cells were obtained from seven patients at baseline using NK cell separation microbeads (Miltenyi Biotech, Germany) and cultured with lamivudine (Moravek Biochemical, Brea, CA, USA) or LdT (Novartis, Basel, Switzerland) at concentration of 5 µg/ml, which is equivalent to effective drug concentrations in the patients’ peripheral blood. Cells were collected at 1, 2, 4, and 8 h, and NKG2A, NKG2D, NKG2C, NKp30, and NKp46 expression was assayed by fluorescence-activated cell sorting (FACS) as described above.

IL-15 expression by these NK cells was assayed by FACS or by confocal microscopy (Carl Zeiss, Thornwood, NY, USA) by placing freshly isolated NK cells onto polylysine-treated slides for 4 h of LdT or lamivudine culture. For FACS, CD3^−^ CD16^+^CD56^+^IL15^+^ NK cells were gated as described above, and the fold increase of IL-15 expression was calculated as (LdT or LAM group − PBS group)/PBS group.

### Statistical analysis

All results are presented as mean ± standard error (SEM). Statistical significance was determined using two-tailed analysis of variance (ANOVA) tests. Correlations between percentages of peripheral NK cells and clinical indices were tested by two-tailed Pearson correlations. All statistical analyses were performed using SPSS v.11 (SPSS, Chicago, IL, USA). *p* value <0.05 was considered statistically significant.

## Results

### Peripheral NK cells recover during antiviral treatment

**Fig. 1:**
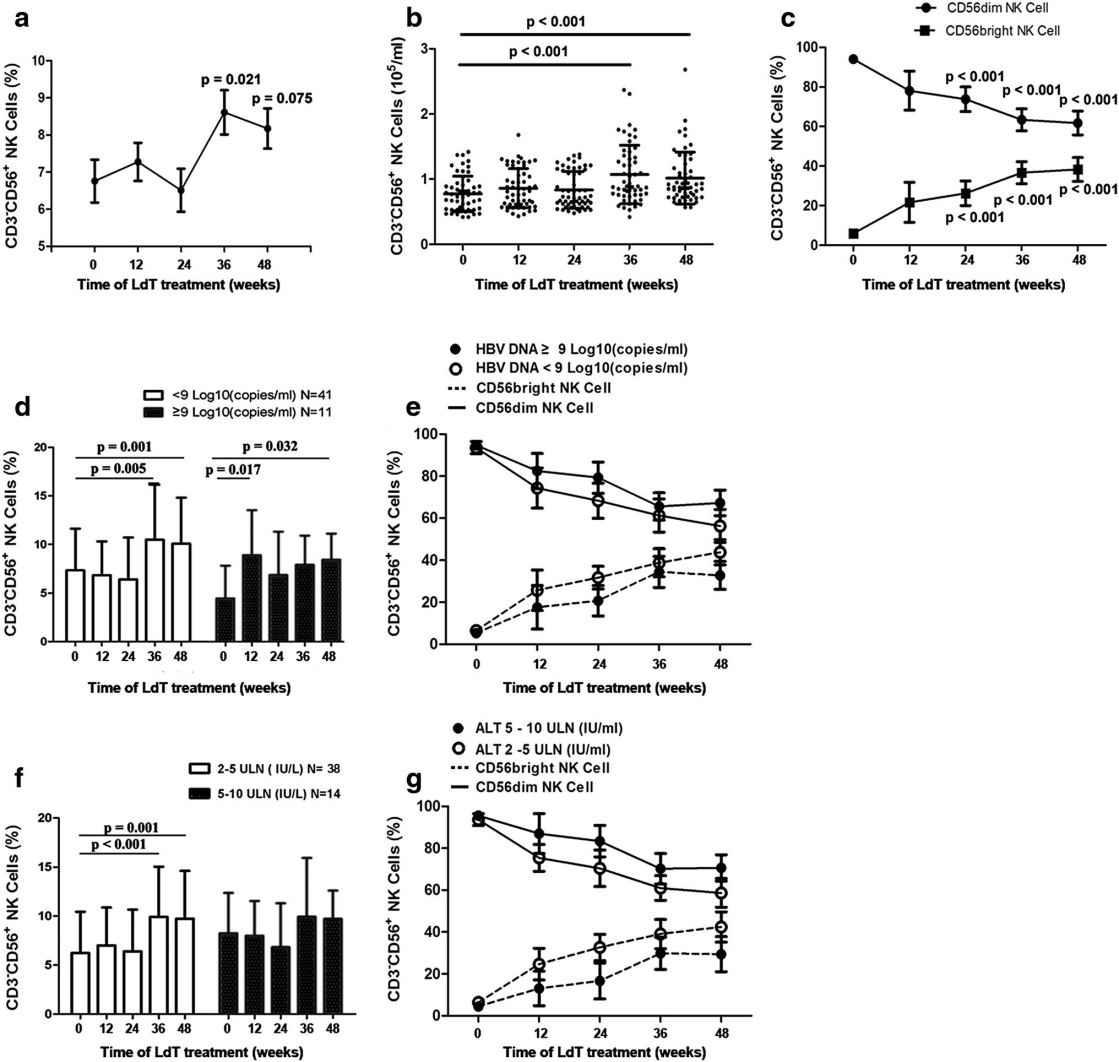
Numbers of peripheral NK cells recovered during antiviral treatment. Both **a** percentage and **b** absolute number of peripheral NK cells significantly increased from week 36 to 48 on treatment. **c** The number of CD56^bright^ NK cells was increased, accompanied with a decline of CD56^dim^ NK cells from week 24 to 48 during LdT treatment when compared with baseline level (*p* < 0.001). **d** Significantly increased peripheral NK cells in patients with low viral load [<9 log_10_ (copies/ml)] at week 36 (*p* = 0.005) and 48 (*p* = 0.001). **e** More elevation of CD56^bright^ NK cells and more decline of CD56^dim^ NK cells were observed in patients with low viral load, but no significant difference was shown between groups with high and low viral load. Patients with baseline ALT 2–5 × the upper limit of normal (ULN) showed better total NK cell (**f**) and CD56^bright^ NK cell (**g**) recovery

Typical representative dot plots of peripheral NK cells are shown in Supplementary Fig. 2. Compared with baseline, the percentage of peripheral NK cells was significantly higher at week 36, decreasing slightly at week 48 (Fig. 1a). The absolute number of NK cells showed a similar trend (*p* < 0.001, Fig. 1b). Notably, the percentage of CD56^bright^ NK cells increased continuously, while that of CD56^dim^ NK cells decreased during LdT treatment (Fig. 1c).

Data from the LdT phase III clinical trial showed that patients with baseline HBV DNA below 9 log_10_ copies/ml or aminotransferase (ALT) 2–5 the upper line of normal (ULN) experienced a higher rate of HBeAg seroconversion [11]. To elucidate the relationship between NK cell changes and immune control (HBeAg seroconversion), we stratified patients by ALT or HBV DNA level to measure the potential difference in numbers and function markers of NK cells. At baseline, NK cell frequencies were comparable in the high (≥9 log_10_ copies/ml) and low (<9 log_10_ copies/ml) HBV DNA groups (*p* = 0.051, Fig. 1d). During treatment, the percentage of peripheral NK cells increased significantly at week 36 and 48 in patients with low viral load (*p* = 0.001 and 0.005, Fig. 1d). However, in patients with high viral load, the percentage of peripheral NK cells was significantly higher at week 12 compared with baseline (*p* = 0.017) but decreased slightly at week 48 (*p* = 0.023, Fig. 1d).

In patients with baseline ALT two- to fivefold higher than ULN, peripheral NK cells were increased at week 36 (*p* = 0.001) and 48 (*p* = 0.005) (Fig. 1f). However, no significant changes in NK cell populations were observed in patients with baseline ALT five- to tenfold higher than ULN.

Patients with baseline levels below 9 log_10_ (copies/ml) or baseline ALT level two- to fivefold ULN (Fig. 1e, g) showed improved CD56^bright^ NK cell recovery compared with those with higher HBV DNA or lower ALT at baseline, but this did not reach statistical significance.

### Activation of peripheral NK cells recovers during antiviral treatment

**Fig. 2:**
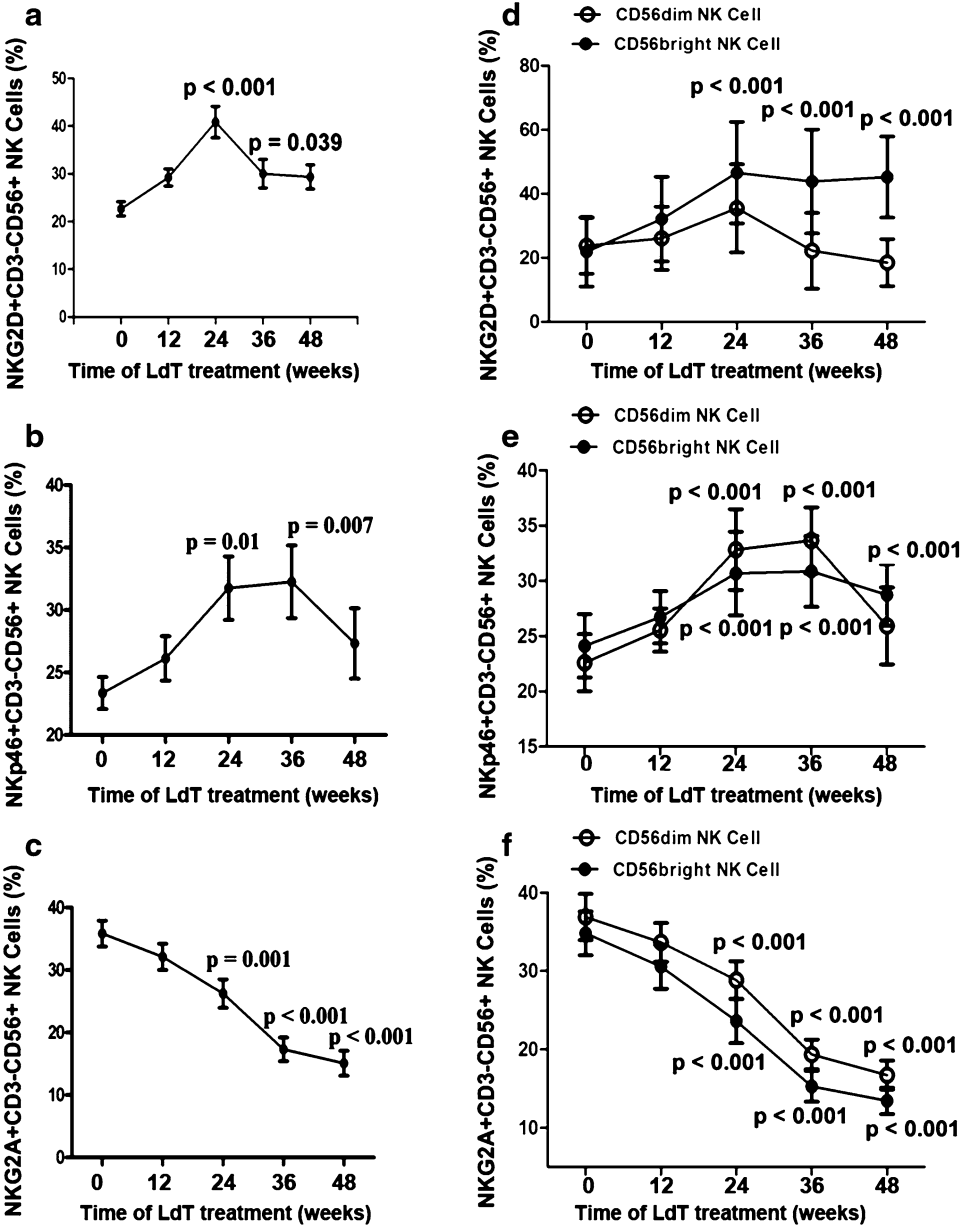
Activation of peripheral NK cells recovered during antiviral treatment. **a** Percentage of NKG2D^+^NK cells was significantly enhanced from week 24 to 48 (*p* < 0.05). **b** Percentage of NKp46^+^NK cells was significantly elevated from week 24 to 36 (*p* = 0.022 and *p* < 0.001), with a sharp decline at week 48. **c** Expression of inhibitory receptor NKG2A was significantly lower in weeks 12 to 48 than at baseline (*p* < 0.001 each). **d** NKG2D^+^CD56^bright^ NK cells, without NKG2D^+^CD56^dim^ NK cells, expanded from week 24 to 48 (*p* < 0.001). **e** Percentages of NKp46^+^CD56^bright^ NK cells and NKp46^+^CD56^dim^ NK cells significantly elevated from week 24 to 36 (*p* < 0.001), and showed a decline at week 48 subsequently. **f** NKG2A^+^CD56^bright^ NK cells and NKG2A^+^CD56^dim^ NK cells significantly decreased from week 24 to 48 (*p* < 0.001)

To evaluate the activation state of peripheral NK cells, their surface expression of inhibition and activating receptors, including NKp30, NKp44, NKp46, NKG2C, NKG2D (activating), and NKG2A (inhibiting), was assayed. The percentages of NK cells expressing NKG2D and NKp46 were significantly higher at week 24 or 36 (Fig. 2a, b), whereas the percentage expressing NKG2A decreased significantly from week 12 to 48 (Fig. 2c). The percentages of cells expressing NKp30, NKp44, and NKG2C remained unchanged (data not shown).

To further explore functions of CD56^bright^/CD56^dim^ NK cells, we measured the expression of activation or inhibitory receptors on both subsets of peripheral NK cells. The NKG2D^+^CD56^bright^ and NKp46^+^CD56^bright^, but not the NKG2D^+^CD56^dim^ or NKp46^+^CD56^dim^ cells, were significantly elevated from week 24 to 48 of LdT treatment (Fig. 2d, e) (*p* < 0.001). NKG2A^+^CD56^bright^ and NKG2A^+^CD56^dim^ NK cells decreased significantly during LdT treatment from week 24 to 48 (Fig. 2f, *p* < 0.001).

### Increased NKG2D^+^CD56^bright^ NK cells correlate with HBeAg seroconversion during antiviral treatment

**Fig. 3:**
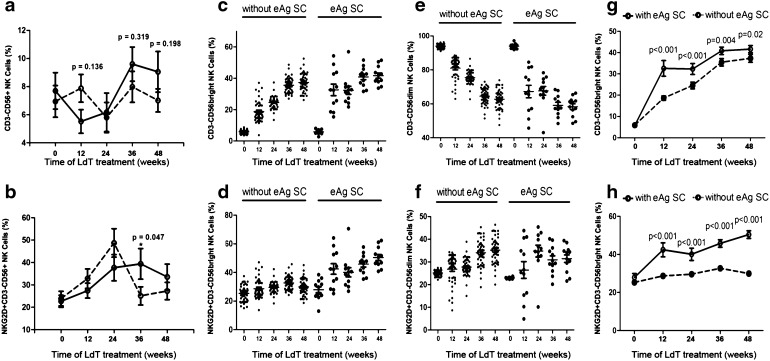
Increased NKG2D^+^CD56^bright^ NK cells correlated with HBeAg seroconversion (eAg SC) during antiviral treatment. **a** Percentage of NK cells in patients with HBeAg seroconversion showed an elevating trend during LdT treatment, but without significance. **b** Expression of NKG2D on peripheral NK cells at week 36 was significantly higher in patients with eAg SC (*p* = 0.047). **c** Percentage of CD3^−^CD56^bright^ and CD3^−^CD56^dim^ NK cells in groups with or without eAg SC shown on scatter plot. **d** Percentage of NKG2D^+^CD3^−^CD56^bright^ and NKG2D^+^CD3^−^CD56^dim^ NK cells in groups with or without eAg SC shown on scatter plot. **e** Patients with eAg SC exhibited significantly higher percentage of CD3^−^CD56^bright^ NK cells from week 12 to 48 compared with the group without eAg SC (*p* < 0.001). **f** Percentage of NKG2D^+^CD56^bright^ NK cells was significantly elevated during LdT treatment in patients with eAg SC (*p* < 0.001)

Based on their significant changes at week 48, the percentages of NK cells and the expression of NKG2D, NKp46, and NKG2A were assayed in patients who showed HBeAg seroconversion (eAg SC) at week 48. The percentage of peripheral NK cells and the expression of NKG2D, NKp46, and NKG2A receptors did not differ significantly between the HBV DNA negative and positive groups at week 48 of LdT treatment (data not show). In HBV DNA negative patients, total NK cells showed a trend to be higher in subjects with HBeAg seroconversion at week 12, 36, and 48 when compared with those without seroconversion (Fig. 3a). NKG2D expression on peripheral NK cells was significantly higher in patients with HBeAg seroconversion at week 36 (*p* = 0.047, Fig. 3b) compared with those without. In contrast, NKG2A and NKp46 expression were similar in patients with and without seroconversion (Supplementary Fig. 3). Patients with HBeAg seroconversion also exhibited a significantly higher percentage of CD3^−^CD56^bright^ (Fig. 3c, e) and NKG2D^+^CD56^bright^ NK cells (Fig. 3d, f) compared with the HBeAg-positive group. The percentage of CD3^−^CD56^dim^ or NKG2D^+^CD56^dim^ NK cells did not show significant differences between these groups at any time point (data not shown).

### Peripheral interleukin 15 is significantly elevated during antiviral treatment

**Fig. 4:**
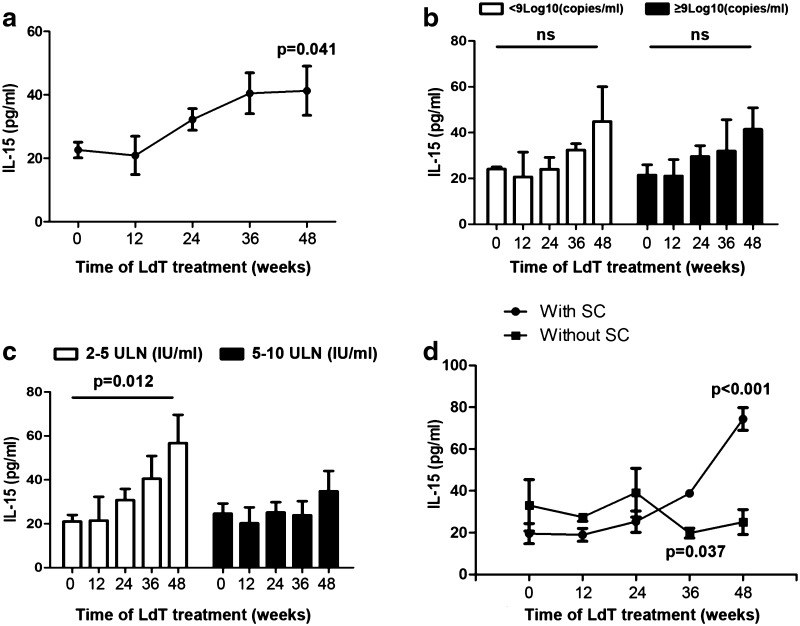
Serum IL-15 level elevated during antiviral treatment. **a** Serum IL-15 significantly elevated at week 48 of LdT treatment (*p* = 0.041). **b** No significance was shown between patients with baseline HBV DNA less than 9 log_10_ (copies/ml) versus more than 9 log_10_ (copies/ml). **c** Patients with baseline ALT 2–5 ULN showed significantly enhanced IL-15 expression at week 48 (*p* = 0.012). **d** Patients with HBeAg seroconversion had higher IL-15 level at week 36 and 48 (*p* = 0.037 and *p* < 0.001, respectively)

To screen for key cytokines, we measured circulating levels of IL-2, IL-4, IL-6, IL-10, IL-15, TNF-α, and IFN-γ at baseline and during LdT treatment. Only IL-15 was significantly elevated during antiviral treatment, starting at week 24 and reaching significance at week 48 (Fig. 4a, Supplementary Fig. 4). While there was no difference between patients with baseline HBV DNA above or below 9 log_10_ (copies/ml), subjects with baseline ALT 2–5 ULN but not ALT 5–10 ULN exhibited significantly enhanced IL-15 expression at week 48 (Fig. 4b, c). Furthermore, patients with HBeAg seroconversion had highly significantly elevated IL-15 levels at week 36 and 48 compared with those remaining HBeAg positive (Fig. 4d).

### Significant increase of NKG2D and IL-15 production in peripheral NK cells treated with LdT in vitro

**Fig. 5:**
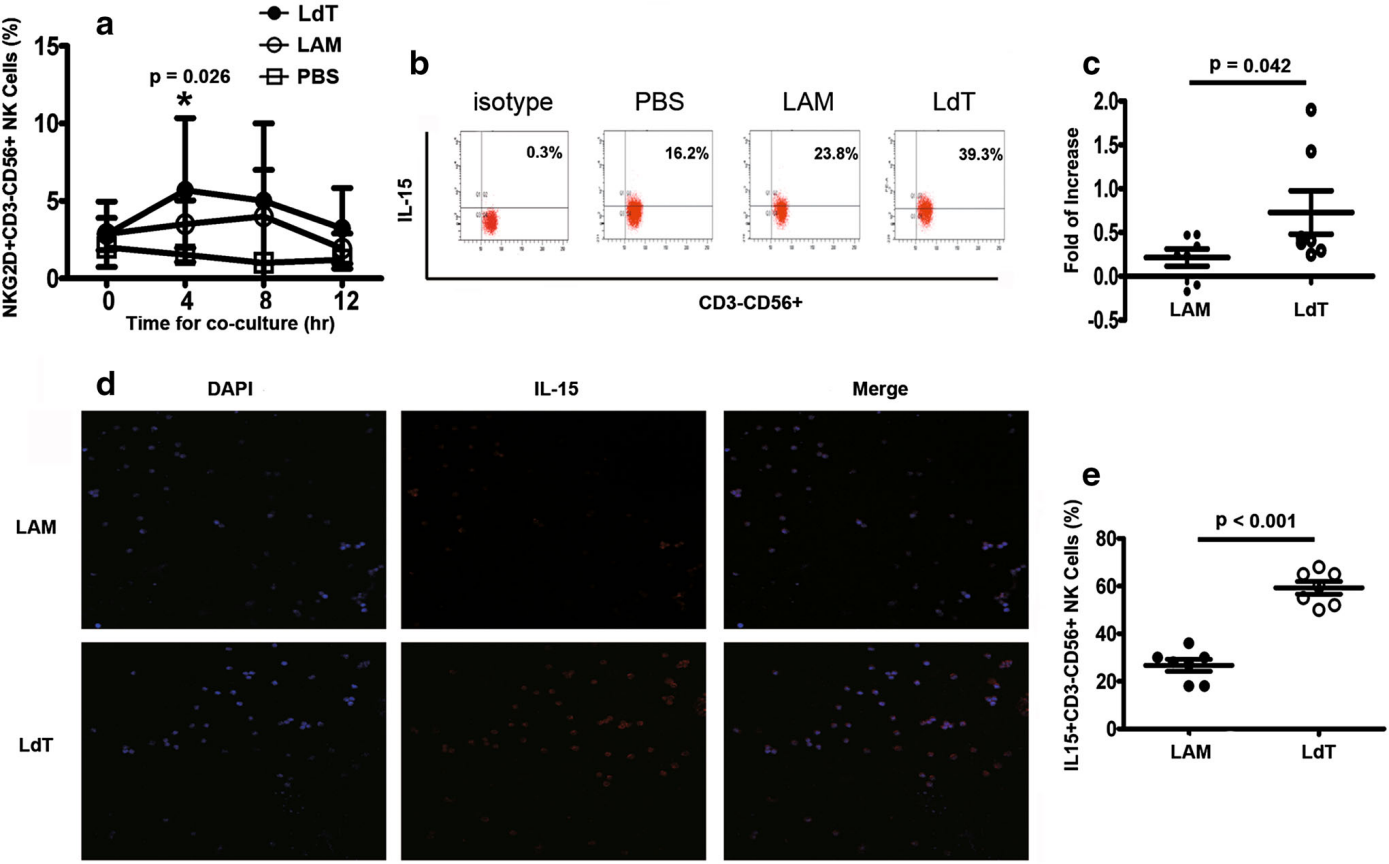
Significant increase of NKG2D and IL-15 production in peripheral NK cells treated with LdT in vitro. Peripheral NK cells that were isolated from seven baseline patients and cultured for 4 h with LdT showed significant increases in NKG2D expression (*p* = 0.026) (**a**) and increased secretion of IL-15 (**b**, **c**, *p* = 0.042). **d** Detection of IL-15 by confocal microscopy in NK cells cultured with LdT. IL-15 was observed mainly in the endochylema (*red*) and was much higher in NK cells cultured with LdT than with lamivudine in semiquantitative assay (**e**, *p* < 0.001)

Peripheral NK cells isolated from HBeAg-positive CHB patients at baseline were incubated with LdT or lamivudine (LAM), and the expression of activating and inhibitory receptors was measured. After 4 h, the expression of NKG2D in cells incubated with LdT was significantly higher than at baseline (*p* = 0.026), an effect lasting for 12 h (Fig. 5a). In parallel, NKG2D expression also increased in LAM-treated NK cells, but the difference did not reach significance (Fig. 5a). There were no significant changes in the expression of other molecules relevant to NK cell modulation, including the inhibitory receptor NKG2A and the activating receptors NKp30, NKp46, and NKG2C, after treatment with either LdT or LAM (data not shown).

To understand the mechanism by which LdT promotes the expression of NKG2D on NK cells, peripheral NK cells isolated from seven treatment-naïve HBeAg-positive CHB patients were cultured with LdT or LAM, and IL-15 expression was assayed. After 4 h, IL-15 expression was significantly higher in cells cultured with LdT than with LAM (Fig. 5b, c, *p* = 0.042). Confocal microscopy showed that IL-15 was expressed mainly in the cytoplasm (Fig. 5d), and immunohistochemical quantitation revealed a significantly increased number of IL-15^+^ NK cells after LdT versus LAM treatment (Fig. 5e, *p* < 0.001).

## Discussion

HBV is intrinsically immunogenic. NUC treatment has largely been reported to affect adaptive immune cells similarly to IFN-α, since they have been shown to induce an increased frequency and effector function of HBV-specific CD4^+^ and CD8^+^ T cells [12, 13]. Thus LdT and LAM both reduced expression of the immune inhibitory molecule programmed cell death (PD)-1 on total T cells and their HBV-specific subsets [14, 15], and LdT has also been associated with enhanced production of effector T cell inducing IL-21 and recovery of follicular helper T cells, which provide functional help to both T and B cells [16]. However, the impact of NUC treatment on innate immune cells is less well defined.

Our data show that the frequency and activation phenotype of CD56^bright^ and NKG2D^+^ NK cells gradually increased during Ldt treatment. However, a recent study showed that the inflammatory phenotype of NK cells declined upon viremia suppression and ALT normalization induced by NUC therapy [17]. This may be because of the character of the patients enrolled, who were HBeAg negative, in contrast to the HBeAg-positive patients in our study. To our knowledge, HBV e antigen is a strong immune inhibitory molecule whose clearance could induce immune system recovery. Additionally, various different NUCs, such as ETV, LAM + ADV, LAM or LAM + TDF, were used for treatment, potentially possessing different immune regulatory roles, as indicated by the comparison between LdT and LAM in our study. Furthermore, the ALT level, longitudinal treatment time, and HBV genotype could result in different host immune situations.

Our data show that LdT beneficially modulates peripheral NK cells to enhance expression of IL-15 and NKG2D. Our findings may be explained as secondary to a rapid decline of the hepatitis B viral load. However, a direct immunomodulatory effect of NUCs has recently been implicated in patients with HBV infection [18]. Better evaluation of such direct immune modulatory effects of NUCs is important, since further attempts to boost HBV-specific immune responses will need to be based on patients in whom HBV replication is suppressed by highly potent NUCs. In line with the relevance of innate immune cells in NUC-induced antiviral effects, we previously reported that LdT not only promotes T-helper 1 cytokine production but also downregulates programmed death ligand 1 (PDL-1) expression in macrophages in a MHV-3 infected hepatitis model [19].

Antiviral NK cell activation depends on the balance between stimulatory and inhibitory signals transmitted by activating and inhibitory receptors. However, little is known about the mechanisms underlying the regulation of the expression of these NK cells. IL-15 has been shown to promote NKG2D-mediated signaling by coordinately increasing the levels of NKG2D and expression of the intracellular adaptor DAP10 via phosphorylation of Erk in a PI3 kinase-dependent manner [20, 21]. Generally, while previous studies showed that the immune activating NKG2D receptor is induced by IL-15 from other cell types, our study suggests that there also exists a para- or autocrine pathway of IL-15-mediated activation of NK cells, which should be further evaluated in ex vivo and in vivo studies using, e.g., IL-15 blocking agents. On the other hand, NK cell recovery appears after 36 weeks of therapy in vivo, which may be not good to be inducted by the effect of Ltd on NK cells’ ability of producing IL-15 in vitro. This may derive from the complexity of the immune balance among diverse immunocytes in vivo, in which innate immune (M1 macrophage) and acquired immune (CD4^+^ T cells) components may play important regulatory roles along with NK cells themselves.

NK cells were mainly divided into two subpopulations (CD56^bright^ and CD56^dim^ NK cell) based on CD56 density. Functionally, immunoregulatory CD56^bright^ NK cells produce abundant cytokines in response to infection, while CD56^dim^ NK cells are inefficient cytokine producers yet they are efficient effectors of natural and antibody-dependent infected cell lysis. Moreover, CD56^bright^ NK cells are powerful producers of interferon, an immunoregulatory cytokine that plays a critical role in clearance of infectious pathogens. The innate immune system is impaired in the chronic phase of HBV infection and quickly recovers in the pathogen clearance phase. In line with other studies, our data showed that the percentage of activation receptor expression on CD56^bright^ NK cells, not CD56^dim^ NK cells, showed better recovery. The enhanced expression of IL-15 may facilitate the expansion of CD56^bright^ NK cells, but not CD56^dim^ NK cells.

In addition, we observed that patients with baseline ALT 2–5 ULN or HBV DNA less than 9 log_10_ (copies/ml) who obtained higher rates of HBeAg seroconversion developed higher numbers and better functional restoration of CD56^bright^ NK cells than patients with higher baseline ALT or higher HBV DNA load. Interestingly, no direct relationship was found between levels of HBV DNA and restoration of NK cell number and activity, resembling data on HBV DNA levels in patients exhibiting higher HBsAg clearance or HBeAg seroconversion under treatment with IFN-α [13]. It was suggested that the antiviral efficacy of Peg-IFN-α in CHB patients may be limited by its depleting effect on CD8^+^ T cells, while on the other hand it induced proliferation, activation, and antiviral potential of CD56^bright^ NK cells via enhanced IL-15 and NKp46 [22]. Along this line, we found elevated IL-15 and natural cytotoxicity receptor (NCR) NKG2D in LdT-treated CD56^bright^ NK cells, which would substantiate a common and synergistic mechanism for the antiviral activity of IFN-α and LdT in NK cells. We could also confirm the hypothesis that LdT may contribute to enhanced IL-15 and NCR receptor expression via promoting endogenous IFN-α expression, based on a small substudy in patients with HBeAg seroconversion (Supplementary Fig. 5). This may be an alternative mechanism of asynchronous NKG2D^+^CD56^bright^ NK cell induction and serum IL-15 levels, as observed in our study. Furthermore, these results may provide a rationale for endogenous IFN-α production as a welcome additive effect of NUC treatment in CHB.

In conclusion, although studies have indicated that the host immune system could be modulated with inhibition of HBV DNA during NUC-based antiviral treatment, the direct host immunomodulation induced by antiviral agents has not yet been explored. Our results firstly demonstrated that functional restoration of CD56^bright^ NK cells contributed to better treatment efficacy via IL-15 and NKG2D during antiviral treatment with LdT. This type of direct immune control role may be supplementary to secondary immune modulation with virus inhibition by NUCs. These results may also contribute to comprehensive understanding of the importance of a restored innate immune system to achieving long-term viral control in patients with CHB.

## Electronic supplementary material

Below is the link to the electronic supplementary material.

Supplementary material 1 (TIFF 247 kb)

Supplementary material 2 (TIFF 212 kb)

Supplementary material 3 (TIFF 6319 kb)

Supplementary material 4 (TIFF 2068 kb)

Supplementary material 5 (TIFF 3842 kb)

## Abbreviations

LdT: Telbivudine
LAM: Lamivudine
HBV: Hepatitis B virus
CHB: Chronic hepatitis B
NK: Natural killer
ALT: Alanine transaminase
HBsAg: Hepatitis B surface antigen
HBeAg: Hepatitis B e antigen
SC: Seroconversion
PBMCs: Peripheral blood monocytes
ULN: Upper limit of normal
IL-15: Interleukin 15
