# Supplementary figures and images for: Functional restoration of CD56bright NK cells facilitates immune control via IL-15 and NKG2D in patients under antiviral treatment for chronic hepatitis B
Source: Hepatol Int. 2017 Jun 20;11(5):419–28. doi: 10.1007/s12072-017-9803-4 (PMC5606950; doi:10.1007/s12072-017-9803-4)

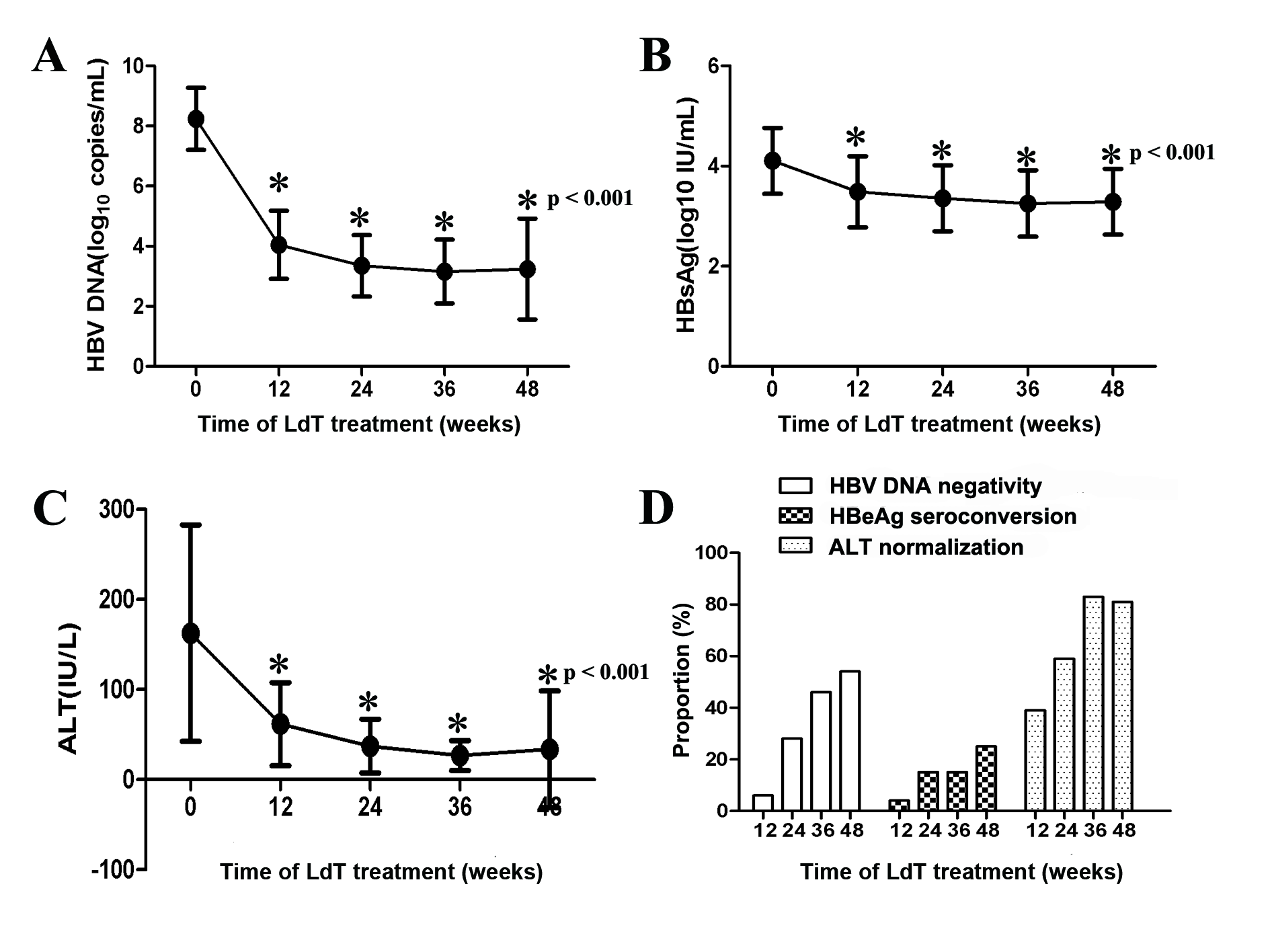

Supplement: Supplementary file 1 — Supplementary material 1 (TIFF 247 kb) [file 12072_2017_9803_MOESM1_ESM.tif]

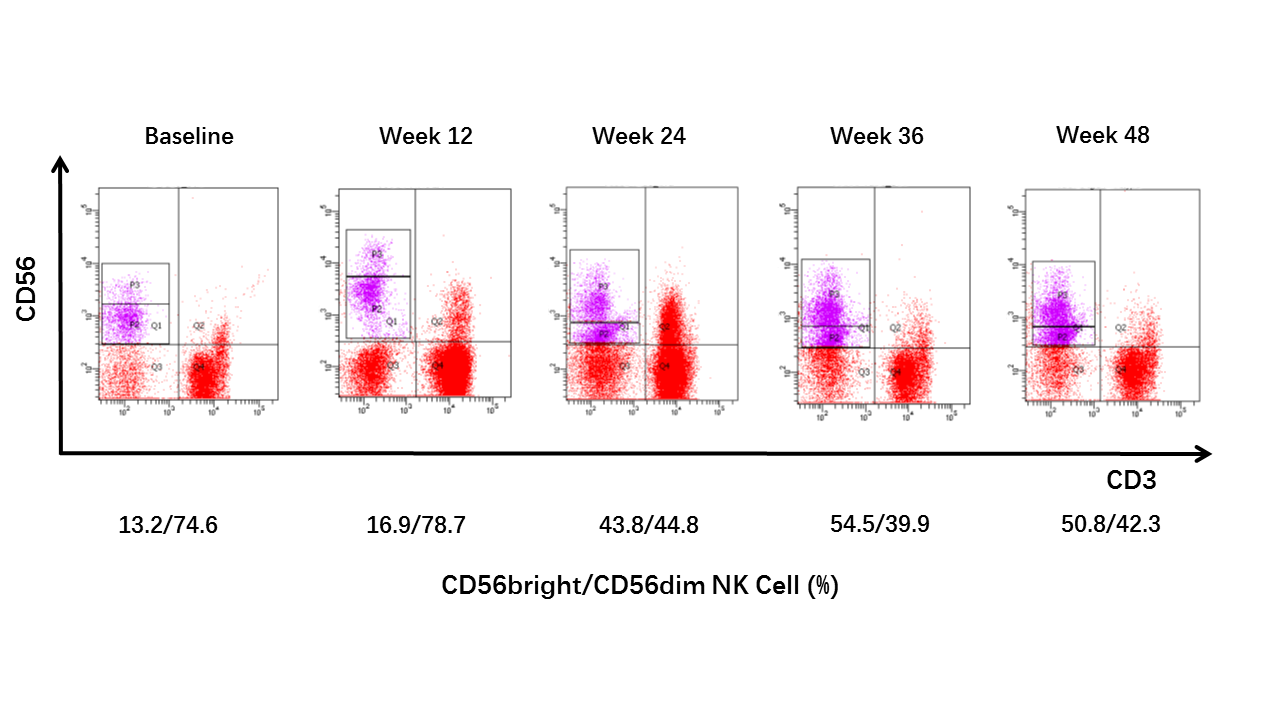

Supplement: Supplementary file 2 — Supplementary material 2 (TIFF 212 kb) [file 12072_2017_9803_MOESM2_ESM.tif]

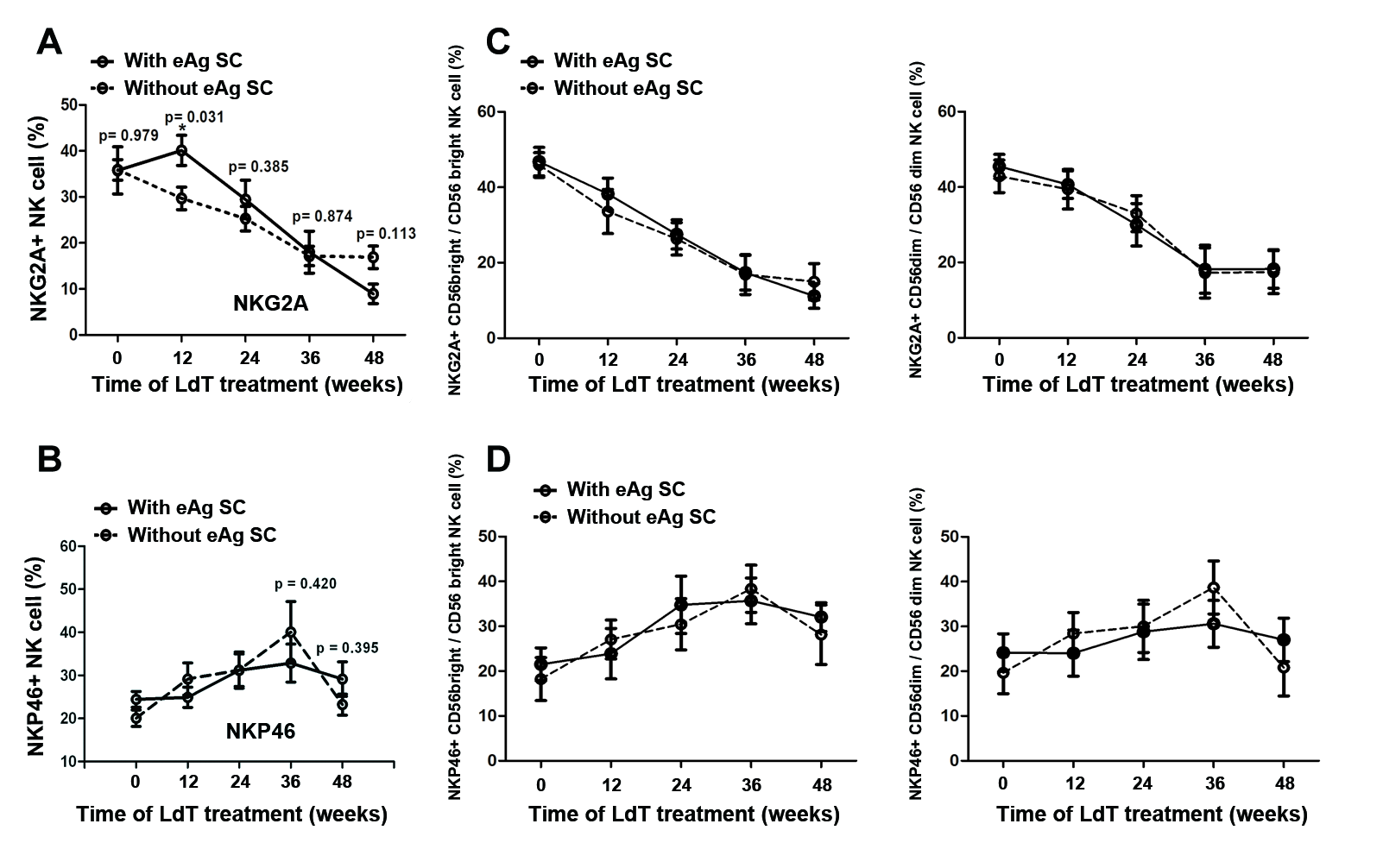

Supplement: Supplementary file 3 — Supplementary material 3 (TIFF 6319 kb) [file 12072_2017_9803_MOESM3_ESM.tif]

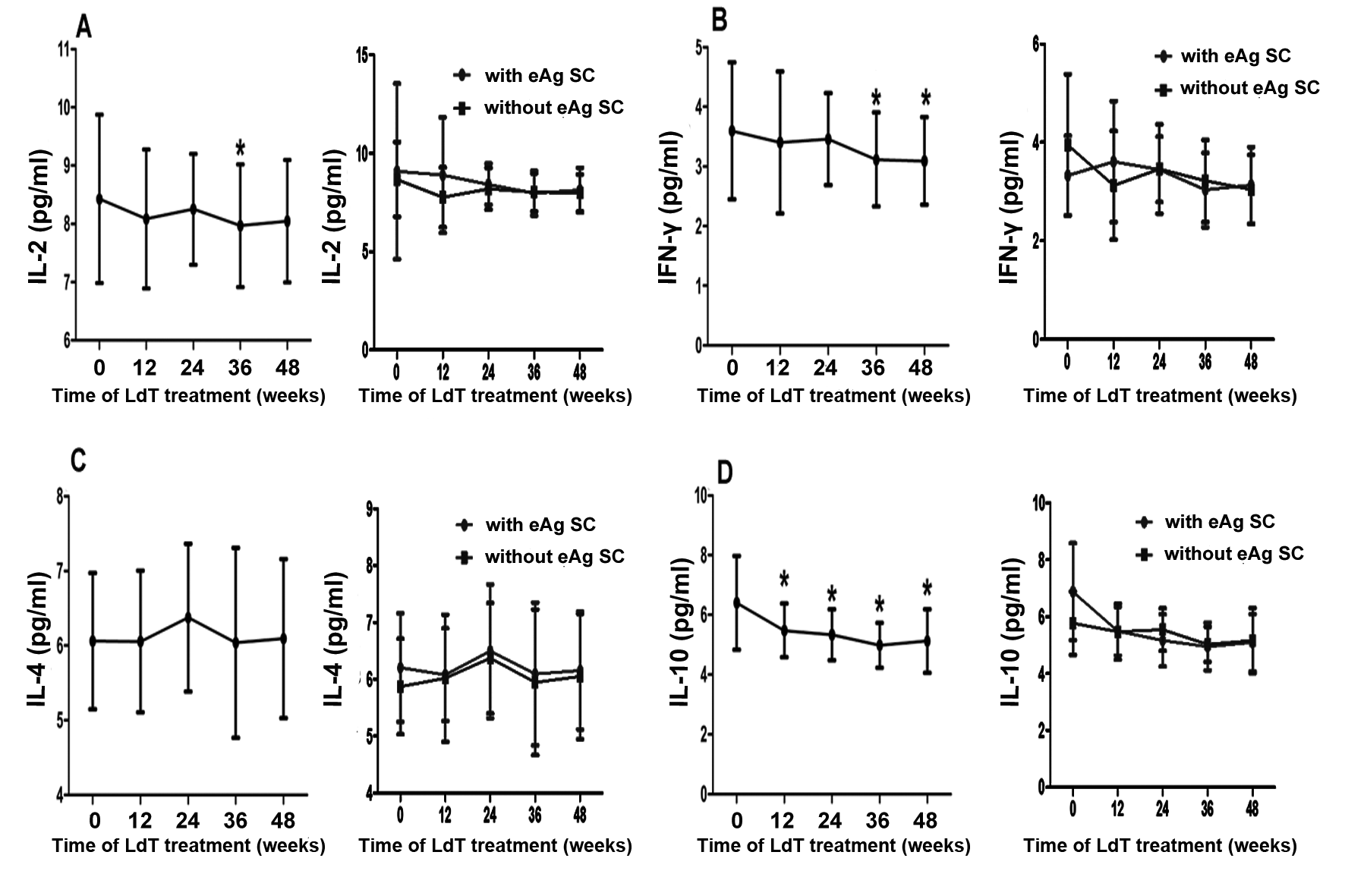

Supplement: Supplementary file 4 — Supplementary material 4 (TIFF 2068 kb) [file 12072_2017_9803_MOESM4_ESM.tif]

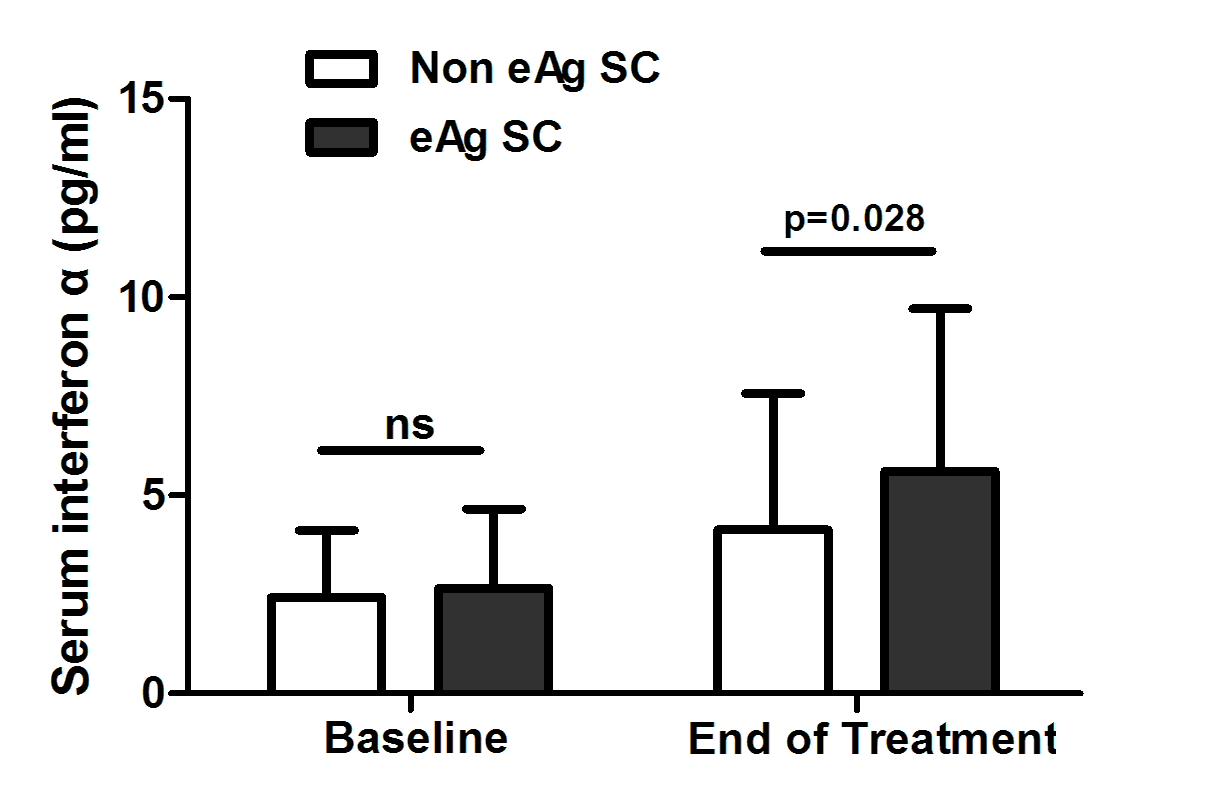

Supplement: Supplementary file 5 — Supplementary material 5 (TIFF 3842 kb) [file 12072_2017_9803_MOESM5_ESM.tif]
